# Supplementary material for: DNA Methylation as an Early Indicator of Aging in Stored Seeds of “Exceptional” Species Populus nigra L
Source: Cells. 2022 Jun 30;11(13):2080. doi: 10.3390/cells11132080 (PMC9265770; doi:10.3390/cells11132080)
Supplement: Supplementary file 1 [file cells-11-02080-s001.zip › cells-1735295-supplementary.pdf]

Supplementary Equation S1. Method of Δ% 5mC calculation.

X = % 5mC at 95% germination calculated by p.dose from GLM model (control)

Y = % 5mC at one of the arbitrary chosen percentage of germination calculated by p.dose from GLM model.

$$Z = Y/X \times 100\% \quad (S1)$$

$$\Delta = 100\% - z \quad (S2)$$

Calculated Δ was plotted on x axis. Germination (%) was plotted on y axis.

Supplementary Table S1. AIC (Akaike information criterion) and coefficients of constructed GLM models for all seeds, non-desiccated seeds at the MC range 12.5–14.5% and desiccated seeds at the MC range 7.6–7.8%.

**Models**	**Null Deviance on Degrees of Freedom**	**AIC**	**Coefficients of GLM Models**			
			**Estimate**	**Std. Error**	**z Value**	**Pr(>|z|)**
All seeds	100.375 on 119	73.605	1.8068	0.3193	5.658	1.53e-08
Non-desiccated	60.186 on 59	41.184	2.4225	0.6083	3.982	6.82e-05
Desiccated	35.396 on 59	34.459	1.5170	0.4155	3.651	0.000261

Supplementary Table S2. Calculated relative DNA methylation level changes (%Δ) based on GLM models.

**Assumed Germination Level**	**All Seeds**	**Non-desiccated Seeds**	**Desiccated Seeds**
95%	0%	0%	0%
90%	10.004%	8.546%	10.071%
80%*	14.792%	12.880%	15.565%
50%	22.976%	18.891%	24.967%
25%	29.462%	24.070%	32.415%
10%	35.948%	29.237%	39.873%
5%	40.359%	32.761%	44.934%

Supplementary Table S3. The relationship between germination (%) and (Δ%) 5mC. Slopes indicate the percentage of germination change when relative (Δ%) 5mC level changes by 1%.

	**All seeds**	**Non-desiccated**	**Desiccated**
Slope of asymptomatic changes [1]	−0.448 (R^2 = 0.9266)	−0.521 (R^2 = 0.9214)	−0.496 (R^2 = 0.8663)
Slope of rapid loss of germination [2]	−3.784 (R^2 = 0.9994)	−4.885 (R^2 = 0.9998)	−3.295 (R^2 = 0.9994)

[1] Assessed between 95% and 90% of germination, [2] Assessed between 80 and 25% of germination.
